# Evaluation of Antibacterial Activity of Aqueous, Ethanolic and Methanolic Extracts *of* Areca Nut Fruit on Selected Bacteria

**DOI:** 10.1155/2021/6663399

**Published:** 2021-04-16

**Authors:** Neda Jam, Reza Hajimohammadi, Parvin Gharbani, Ali Mehrizad

**Affiliations:** ^1^Department of Chemical Engineering, Ahar Branch, Islamic Azad University, Ahar, Iran; ^2^Department of Chemistry, Ahar Branch, Islamic Azad University, Ahar, Iran; ^3^Department of Chemistry, Tabriz Branch, Islamic Azad University, Tabriz, Iran

## Abstract

Today, the tendency to use of natural preservatives to increase food security has expanded. In the present study, antibacterial effects of Areca Nut fruit extracts were evaluated against *Staphylococcus aureus*, *Escherichia coli*, *Salmonella enterica*, and *Enterobacter aerogenes* bacteria using agar disc diffusion technique. Methanol, ethanol, and water were used as solvents for extraction by maceration method, and extracts were analyzed by GC-MS. The antibacterial activity was evaluated using microtiter broth dilution method to determine minimum inhibitory concentration (MIC) and minimum bactericidal concentration (MBC). Results revealed that all ATCC strains were significantly inhibited by ethanolic and methanolic extracts. Escherichia coli produced a significantly larger zone of inhibition for Gentamicin (35 ± 0.65 mm) and Penicillin (25 mm ± 0.56), while *Enterobacter aerogenes* produced smaller zone of inhibition for Gentamicin (20 ± 0.87 mm) and Penicillin (15 ± 0.87 mm). Also, methanolic extract had considerable antibacterial activity with MIC value of 1.56 mg/mL against *Escherichia coli*. All of extracts were used to evaluate antibacterial effects in prepared cake, and as a result, all pathogenies were the most sensitive by methanolic extract in 100 mg/L of concentration except *Escherichia coli* that were more sensitive by ethanolic extract. In conclusion, the Areca Nut fruit extracts may be used as a natural preservative in food industries. Future studies should focus on the effect of Areca Nut fruit extracts in bakery and drinking industries.

## 1. Introduction

Currently, employing plant sources as an alternative to chemical additives in medicines and foods is growing [1, 2]. Antimicrobial, antioxidant, and anticancer impacts of herbal extracts and essences have drawn a considerable attention in food and pharmaceutical industries for years [3]. Herbal extracts and their components have been known to possess antimicrobial properties [4], which among them we can mention a plant known as Areca Nut [5, 6]. The Areca Nut is the fruit of the areca palm which grows in much of the tropical Pacific, Southeast and South Asia, and parts of east Africa. It is commonly referred to as betel nut, too [7, 8]. Nutrient content of Areca Nut is carbohydrate, lipid, fibre, polyphenol and alkaloid 0.3 to 0.6%, tannin of 15%, and fat of 14% [9]. All parts of the Areca Nut such as husks, shoots, buds, leaves, and roots are used in medicinal [9]. Chemical compounds including Isoguvocine, Choline, Alkaloids, Arecoline, Acecainide, Arecoline, and Guanine have been found in Areca Nut extract [10]. Fruits of this plant include Catechin alpha and seeds contain Arecodin, Arecatine, Beta-Sitosterol, about 15% tannin, approximately 14% fatty acids, little sucrose, Mannan, and Galactan [11, 12]. Catechins are produced by plant naturally and are a metabolic secondary from tannin [13] and have antioxidant and antimicrobial properties. Tannin in Areca Nut exhibits antibacterial and antifungal activities, too [10, 14, 15]. Seeds contain at least four alkaloids, including Arecaidine, Arecoline, Guvacine, and Guvacoline, which among them Arecoline and Guvacoline are the most important components. Both ingredients enhance natural elasticity and peristaltic wave observed in the intestines, without any damage to circulatory and respiratory systems [16]. Antibacterial effect of Areca Nut leaves extract against B. cereus and P. fluorescens was reported [17]. Also, its seed could inhibit the growth and propagation of S. mutans [9]. Areca Nut is known as a powerful antibacterial and antioxidant due to its content of tannin (15%) [9, 18, 19].

Since Areca Nut fruit has been used for centuries with no significant side effects [10], this study was aimed to evaluate of antibacterial effect of methanolic, ethanolic, and aqueous extracts of Areca Nut against *Staphylococcus aureus*, *Escherichia coli*, *Salmonella enterica*, and *Enterobacter aerogenes* pathogens. Since mentioned pathogens are the main pathogens found in bakery products [20, 21], extracts efficiency was investigated as natural antimicrobial food preservatives in cake instead of artificial ones.

## 2. Materials and Method

### 2.1. Materials

Areca Nut's fruit was obtained from the Research and Development unit of Mers herbal pharmaceuticals, Tabriz, Iran, and was stored in a dark place at 4°C.

### 2.2. Chemicals and Growth Medium

Brain heart infusion (BHI) broth medium, blood agar medium, plate count agar medium, medium nutrient broth, Mueller Hinton broth medium, normal saline, glycerin, phosphate-buffered saline (PBS), sheep blood defibrinated, 0.5 MC Farland tube, ethanol, and methanol. All of the chemicals were purchased from Merck Company. Gentamicin and Penicillin were purchased from Sigma Aldrich company.

### 2.3. Bacterial Strains

The used strains were (ATCC 6538) *Staphylococcus aureus*, *Escherichia coli* (ATCC 25922), *Enterobacter aerogenes* (ATCC 13048), and *Salmonella enterica* (ATCC 9270). All were prepared from the Pasteur Institute of Iran and were stored at 4°C.

### 2.4. Preparation of Extracts

After washing, the collected plants were dried in a freeze dryer for 24 hr and, then, ground to fine powder by an electric blender and sieved (mesh 25). The powdered plants were dried again in an oven at 40° C for 24 hr and then used to preparation of extract. Extracts were prepared by dissolving 2 g of the powder in 50 mL of methanol (80%), ethanol (80%), and distilled water to obtained methanolic, ethanolic, and aqueous extracts of Areca Nut, respectively. It was mixed vigorously for 2 min and concentrated at 23°C for 24 h in a laboratory water bath. The mixtures were then filtered through a 0.45 *μ*m filter, concentrated by a rotary evaporator, and dried at 40°C. The extracts were stored at 4°C in dark glass bottles.

### 2.5. Preparation of Bacterial Culture

At first step, BHI Broth was prepared according to the manufacturer's protocol. Then, 0.5 mL of lyophilized cultures was added to 4 cc BHI broth medium and incubated for 18-16 h at 37°C and was repeated for two times. From the first culture, 1-2 drops were inoculated on blood agar medium and potato dextrose agar (PCA) medium and were spread over the surface of the solid medium. Petri dishes were incubated at 37°C for 24-48 h. The second culture was used to prepare stock. Accordingly, nutrient broth medium and sterile 18% glycerol were mixed at a ratio of 1 : 5, transferred in 500 *μ*l volumes to Eppendorf microtubes, and stored at -20°C. After a few days, prepared cultures were transferred to BHI broth medium and after 16-18 h were revived for analysis. To check the purity of the cultures, the bacteria were cultivated in blood agar medium. In order to perform a disk diffusion (DD) experiment, bacterial suspensions by turbidity of the 0.5 McFarland were prepared, and the inoculum was provided directly from the distinct colonies.

### 2.6. Dilution

Dry extracts were diluted with sodium phosphate buffer solution (pH = 7.2,100 mg/mL) and were shaken vigorously for 2 min by a vortex mixer. Homogenized mixtures were filtered through a 0.22-*μ*m-pore-size membrane filter.

### 2.7. Agar Disk Diffusion Assay

Agar well diffusion method is widely used to evaluate the antimicrobial activity of plants or microbial extracts [22]. The antibacterial activity of extracts was evaluated using the disk diffusion method. 20 mL of suspension containing bacteria was spread on Mueller Hinton agar. After drying agar culture plates, paper discs containing 100 mg/mL of extracts were evenly distributed over the surface of the plates. Then, the wells were punched over the plates of 6.4 mm diameter, and each extract was poured into separate wells. Plates soaked with Gentamicin and Penicillin (0.006 mg/mL) were used as positive control, and a plate soaked with pure solvent of aqueous solution was used as a negative control. Then, plates were incubated at 37 ± 2°C for 24 h. After incubation, the diameter of growth inhibition zone was measured in mm and recorded. Experiments were repeated three times.

### 2.8. Minimal Inhibitory Concentration (MIC) and Minimal Bactericidal Concentration (MBC)

Minimal inhibitory concentration (MIC) and Minimal bactericidal concentration (MBC) were measured using the macrobroth dilution method [23]. Firstly, the cultures were incubated overnight and were diluted. A series of 10 test tubes were used to determine MIC (for each extract and for each studied bacterial species). 8 tubes containing Mueller Hinton Broth were employed for each one. Then, 1 mL of microbial suspensions and different dilutions (1, 10, 100 mg/mL) of the extract were added to each tube. Two tubes consisting of 9 mL of culture medium with 1 mL of diluted extract as a positive control and 9 mL of culture medium with 1 mL of bacterial suspension as a negative control were prepared. All tubes were incubated at 37°C for 24 h. After incubation time, tubes were examined for turbidity caused by the inoculated bacteria. Experiments were repeated three times for each extract and each species. A sampling of all the tubes with no bacterial growth was performed, and the samples were cultivated using the pour plate procedure to determine MBC. So, a 1 mL suspension of each tube was mixed with 20 mL of Muller Hinton agar at 48°C in petri dishes. After a while, when agar solidified, plates were incubated for 24 h and tested for microbial growth. The lowest concentration of the extract at which no bacterial growth that was detected was regarded as MBC.

### 2.9. Preparation of Cake

Eggs and sugar were mixed together; oil was added and mixed. Milk and Areca Nut fruit extract were mixed together and were added to the mixture and mixed well. Half the flour was added and mixed for 1 minute. In the end, baking powder and the rest of the flour were added and mixed for 1 min. It was poured into a mold and baked for 20 min at 180°C. Cakes were packed in heat-sealed polyethylene packages and were kept at room temperature until later analysis.

### 2.10. Microbiological Analysis of Cakes

Depending on the components, each food may include a wide variety of microorganisms. Among them, some are the most important species in cake and confectionery industry, so microbiological testing is necessary to detect and identify them. The antibacterial activity of the solvent extracts of Areca Nut fruit was evaluated against some pathogens responsible for foodborne illness consisting of *Staphylococcus aureus*, *Escherichia coli*, *Salmonella enterica*, and *Enterobacter aerogenes* pathogens in cake. Cakes containing solvent extracts (three various concentrations) were prepared and assessed in 1^st^, 30^th^, 60^th^, and 80^th^ days. A cake without no extract was employed as control. A sterile cutter was applied for sampling from the surfaces, borders, underneath, and inside the sample. The specimens were put in a porcelain mortar (sterilized by heating) and were blended well, milled, and homogenized. 1 gram of specimen was placed into a sterile tube consisting of 9 cc of physiological serum (0.1 g/cc). Different concentrations of the sample (0.01, 0.001 g/cc) were prepared. 0.1 cc of each dilution was removed by pipette and distributed uniformly on the surface of potato dextrose agar (PCA) medium by using standard hockey stick spreaders. Plates were stacked, fixed, and incubated at 37°C for 48 h. The colonies were counted to determine the number of CFU, and the results were recorded.

### 2.11. GC-MS Analysis

GC-MS analysis of the extract of Areca Nut fruit was performed using a Shimadzu GC-17A system comprising a Gas Chromatograph interfaced to a Mass Spectrometer (GC-MS) equipped with an Agilent J&W (100% Dimethylpolysiloxane) fused a capillary column (30 m, 0.32 mm, 1.00 *μ*m) and FID detector. Helium gas was used as a carrier gas at a constant flow rate of 5 mL/min. The oven temperature was programmed from 70°C (for 2 min), with an increase to 300°C (30 min). Mass spectra were taken at 70 eV. The solvent delay was 3 min, and the total GC/MS running time was 78 min.

## 3. Results and Discussion

### 3.1. GC-MASS Analysis of Areca Nut Fruit Extracts

**Figure 1:**
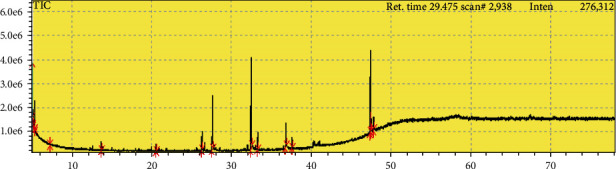
GC/MS chromatogram of Areca Nut extracts.

GC-MASS analysis of Areca Nut fruit extracts is shown in Figure 1 and Table 1. Results showed six peaks which indicate the presence of 6 phytochemical constituents that is identified. As Table 1, Cyclohexane, Heptane, 2,4-bis(1,1-dimethylethyl)-Phenol, Lauric acid, Tetradecanoic acid, and Diethylhexylphthalate are the main constituents of Areca Nut fruit extracts.

### 3.2. Antibacterial Activity of Areca Nut Extracts

The test of antibacterial activity is carried out by measuring the clear zone developed in petri dish. The results showed that all extracts were effective in inhibiting the growth of bacteria except aqueous extract against *Enterobacter aerogenes* and *Staphylococcus aureus* bacteria (Table 2). The diameter of the bacterial growth inhibition zone in control samples (Penicillin and Gentamicin) was larger than the inhibition zone of the extracts. Escherichia coli produced a significantly larger zone of inhibition for Gentamicin (35 ± 0.65 mm) and Penicillin (25 mm ± 0.56) than others, while *Enterobacter aerogenes* produced a smaller zone of inhibition for Gentamicin (20 ± 0.87 mm) and Penicillin (15 ± 0.87 mm). Produced inhibition zone by *Staphylococcus aureus* and *Salmonella enterica* for Gentamicin were 25 ± 0.54 and 30 ± 1.01 mm and for Penicillin were 20 ± 0.96 and 18 ± 0.98 mm, respectively. *Enterobacter aerogenes* were significantly inhibited by methanolic (12 ± 0.87) and ethanolic (10 ± 0.23) extracts, while there is no inhibition zone for aqueous extract. Furthermore, our findings disclosed that the extracts were more effective against *Escherichia coli* and *Salmonella enterica* as gram-negative bacteria and *Staphylococcus aureus* as a gram-positive bacterium. However, extracts showed a good zone of inhibition against the growth of gram-positive bacterium due to the presence of outer membrane of gram-negative bacteria and the enzymes of the periplasmic spaces which act as a barrier against numerous antibiotic molecules and can break down foreign molecules, respectively [22]. According to results, Areca Nut is rich in tannins, terpenoids, alkaloids, and flavonoids [22], and tannin in Areca Nut is effective to inhibit gram-positive bacteria. Tannin binds with peptide on peptidoglycan component from cell walls which in turn disturb the integrity of bacterial cell walls, which cause bacterial cell damage [24, 25]. Finally, it led to disturbance of metabolism process and subsequently the death of cells [26]. It can be deduced that methanolic extracts showed more response to inhibit the growth of all pathogens. The presence of different bioactive chemical agents in the extracts may be due to the antibacterial properties of plants. In this study, extracts showed a better antibacterial activity in results of containing tannins. Maybe tannins have the ability to inactivate several enzymes, microbial adhesion, and cell envelope transport proteins [27]. The statistical results of disc diffusion analysis presented the significance for all pathogens (Table 3). The extracts showed a good zone of inhibition (*p* < 0.05) against all of pathogens.

### 3.3. Results of MIC and MBC

To evaluate the effectiveness of the extracts to inhibit the growth of *Staphylococcus aureus*, *Escherichia coli*, *Salmonella enterica*, and *Enterobacter aerogenes*, MIC assay was employed. Low-MIC and high-MIC values indicate high and low activity of extracts against pathogens, respectively [27]. As a result, the methanolic extract had a considerable antibacterial activity with MIC value of 1.56 mg/mL against *Escherichia coli*. According to, the MIC value of the extracts agreed with their corresponding antibacterial activities. The results of MIC revealed that methanolic and ethanolic extracts have antibacterial activities against all pathogens, while aqueous extracts have a lower antibacterial effect due to the existence of nonpolar molecules in the extracts. As known, the polar components cannot dissolve nonpolar components and hence, antibacterial activities of aqueous extract are decreased. Since methanol has a high potential to dissolve all polar and nonpolar molecules, the methanolic extract showed higher inhibition against the pathogens, especially *Escherichia coli* (1.56 mg/mL). Also, methanolic Areca Nut fruit extract displayed the lowest MBC against *Staphylococcus aureus* and *Enterobacter aerogenes* )3.12 mg/mL(. In this survey, the methanolic extract showed more acceptable effects against the bacterial strains compared with aqueous extract. It can be attributed to the presence of antibacterial agents with different polarities in Areca Nut extract or in other word difference in the solvent efficiency to isolate biologically active compounds. Moure et al. reported that antioxidant and antibacterial properties also depend upon the extraction location and applied techniques [28]. The type of solvent is also one of the most important factors in the isolation of antibacterial and antioxidant substances, as these agents have different polarities [29]. Contrary to our findings, Rahman et al.'s study indicated that methanolic Areca Nut fruit extract substantially inhibited *Staphylococcus aureus* growth at 20 mg/mL concentration, while displayed no growth inhibitory effect on *Escherichia coli*. [30]. Moreover, Ghanwate et al. reported that using disk diffusion approach, aqueous extract of Areca Nut fruit at the concentration of 100 mg/mL inhibited bacterial growth of *Escherichia coli*, *Salmonella enterica*, and *Staphylococcus aureus* with similar efficiency (similar inhibition zone sizes) [31].

### 3.4. Antibacterial Activity of Areca Nut Fruit Extracts in Cake

**Figure 2:**
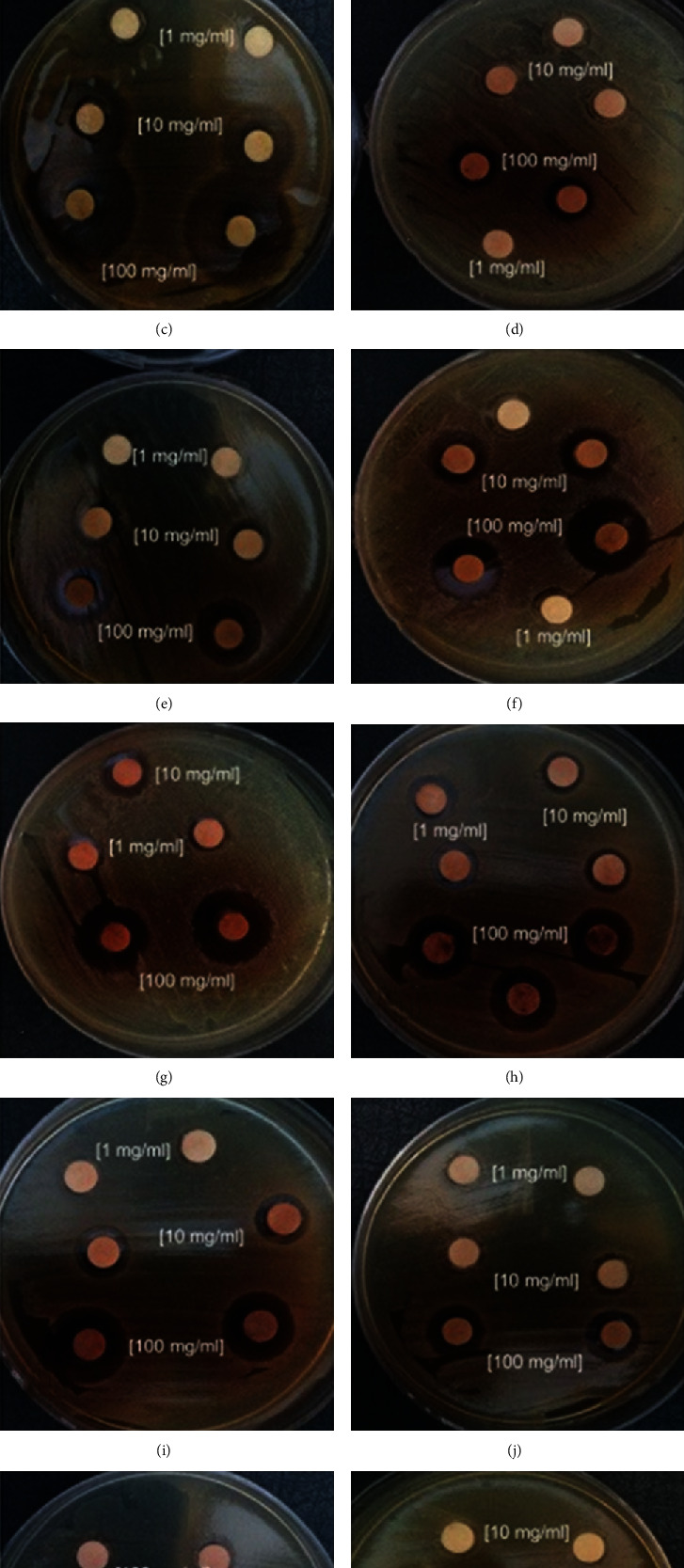
Inhibitory activity of Areca Nut extracts on *Staphylococcus aureus*, *Escherichia coli*, *Salmonella*, and *Enterobacter aerogenes* in various solvents by disc diffusion method, *Escherichia coli* (a) aqueous, (b) ethanolic, and (c) Methanolic; *Staphylococcus aureus* (d) aqueous, (e) ethanolic, (f) methanolic; *Salmonella enterica* (g) aqueous, (h) ethanolic, and (i) methanolic; and *Enterobacter aerogenes* (j) aqueous, (k) ethanolic, and (l) methanolic.

The antibacterial activity of different concentrations of methanolic, ethanolic, and aqueous extracts of Areca Nut fruit against selected pathogens in cake was studied, and results are presented in Table 4 and Figure 2. Based on the inhibition zone diameter (IZD), the ethanolic extract of Areca Nut fruit was effective in growth control of the *Escherichia coli* (20 ± 0.34 mm, 100 mg/mL), *Staphylococcus aureus* (16 ± 0.43 mm, 100 mg/mL), *Salmonella enterica* (16 ± 0.12 mm, 100 mg/mL), and *Enterobacter aerogenes* (15 ± 0.23 mm, 10 mg/mL). The methanolic extract of Areca Nut fruit had a maximum level of inhibition zone against *Escherichia coli* (19 ± 0.23 mm, 100 mg/mL), *Staphylococcus aureus* (17 ± 0.65 mm, 100 mg/mL), *Salmonella enterica* (16 ± 0.76 mm, 100 mg/mL), and *Enterobacter aerogenes* (16 ± 0.34 mm, 1 mg/mL). Aqueous extract of Areca Nut fruit had a maximum level of inhibition zone against *Escherichia coli* (15 ± 0.23 mm, 100 mg/mL), *Staphylococcus aureus* (10 ± 0.45 mm, 100 mg/mL), *Salmonella enterica* (15 ± 0.65 mm, 100 mg/mL), and *Enterobacter aerogenes* (12 ± 0.65 mm, 100 mg/mL). As a result, methanolic extract of Areca Nut fruit was the most effective antibacterial agent against all pathogens at 100 mg/mL concentration. However, aqueous extract of Areca Nut fruit was the least effective antimicrobial agent, and it did not produce antibacterial activity against *Staphylococcus aureus* and *Escherichia coli* bacteria at low concentration. Pour plate counts of four main pathogens in cakes including Areca Nut extracts at 1^st^, 30^th^, 60^th^, and 80^th^ days of baking did not show any observable colonies, whereas the control samples showed the count of 1.12 × 10^3^ cfu/g for each pathogen.

## 4. Conclusion

Methanolic fruit extract of Areca Nut showed an antibacterial effect on both gram-positive and gram-negative bacteria. In addition, results disclosed that the extracts were more effective against *Escherichia coli* as a gram-negative bacterium when compared with other pathogens. For *Escherichia coli*, a minimum inhibitory concentration (MIC) of 1.56 mg/mL was established. Also, the antibacterial activity of Areca Nut fruit extracts in a baked cake revealed that methanolic extract has the most effective antibacterial agent against all pathogens at 100 mg/mL concentration. Additionally, the cakes containing Areca Nut fruit extract displayed acceptable and promising results in terms of technological, organoleptic, and rheological characteristics of the specimens, so that the cakes were approved regarding sensory attributes, notably texture, flavor, and smell. Therefore, our study highlights the potential use of these extracts as natural additives in cake industry to satisfy consumers' needs to use natural preservatives in foods and enhance food safety. Further evaluation of Areca Nut fruit on antiracial activity of other main pathogens in food and bakery industries will be highly effective in the formulation of policies for the management of food safety.

## Figures and Tables

**Table 1:** GC–MS analysis of Areca Nut extracts.

No.	RT	Name of component	Molecular formula	Mw
1	5.039	Cyclohexane	C6H12	84
2	5.362	Heptane	C7H16	100
3	26.30	2,4-bis(1,1-dimethylethyl)-Phenol	C14H22O	206
4	27.61	Lauric acid	C12H24O2	200
5	32.46	Tetradecanoic acid	C14H28O2	228
6	47.44	Diethylhexylphthalate	C24H38O4	390

**Table 2:** Inhibitory activity of Areca Nut fruit extracts on *Staphylococcus aureus*, *Escherichia coli*, and *Salmonella enterica* and *Enterobacter aerogenes* in various solvents by disc diffusion method.

Solvent	Concentration (mg/mL)	Inhibitory activity of Areca Nut fruit extracts (mm)			
		*Enterobacter aerogenes*	*Salmonella* enterica	*Escherichia coli*	*Staphylococcus aureus*
H₂O	100	—	15 ± 0.34	15 ± 0.45	—
C_2_H_5_OH	100	10 ± 0.23	15 ± 0.23	17 ± 1 + 01	16 ± 0.34
CH_3_OH	100	12 ± 0.65	16 ± 1.34	19 ± 087	17 ± 0.4
Gentamicin	0.006	20 ± 0.87	30 ± 1.01	35 ± 0.65	25 ± 0.54
Penicillin	0.006	15 ± 0.87	18 ± 0.98	25 ± 0.56	20 ± 0.96

**Table 3:** Result of significance test (*p* < 0.05) for the mean of the zone of inhibition at two solvents.

Statistical analysis						
Bacteria	Ethanol			Methanol		
	t-value	*p* value	Significant	t-value	p-value	Significant
*Enterobacter aerogenes*	4.35	0.007	Yes	3.87	0.002	Yes
*Salmonella* enterica	3.23	0.0065	Yes	4.54	0.034	Yes
*Escherichia coli*	4.32	0.004	Yes	3.99	0.002	Yes
*Staphylococcus aureus*	2.54	0.001	Yes	3.1	0.004	Yes

**Table 4:** Antibacterial activity inhibition zone diameter (IZD), mm of Areca Nut fruit extracts on *Staphylococcus aureus*, *Escherichia coli*, *Salmonella enterica*, and *Enterobacter aerogenes* in various solvents by disc diffusion method.

*Escherichia coli*			*Staphylococcus aureus*			*Salmonella enterica*			*Enterobacter aerogenes*		
H2O a	Conc. mg/mL	IZD mm	H2O d	Conc. mg/mL	IZD mm	H2O g	Conc. mg/mL	IZD mm	H2O j	Conc. mg/mL	IZD mm
	100	15 ± 0.23		100	10 ± 0.45		100	15 ± 0.65		100	12 ± 0.65
	10	7 ± 0.34		10	—		10	11 ± 0.63		10	10 ± 0.24
	1	—		1	—		1	8 ± 0.34		1	10 ± 0.12

EtOH b	Conc. mg/mL	IZD mm	EtOH e	Conc. mg/mL	IZD mm	EtOH h	Conc. mg/mL	IZD mm	EtOH k	Conc. mg/mL	IZD mm
	100	20 ± 0.34		100	16 ± 0.43		100	16 ± 0.12		100	8 ± 0.11
	10	18 ± 0.23		10	10 ± 0.42		10	11 ± 0.34		10	15 ± 0.23
	1	12 ± 0.98		1	2 ± 0.65		1	12 ± 0.54		1	12 ± 0.14

MeOH c	Conc. mg/mL	IZD mm	MeOH f	Conc. mg/mL	IZD mm	MeOH i	Conc. mg/mL	IZD mm	MeOH l	Conc. mg/mL	IZD mm
	100	19 ± 0.23		100	17 ± 0.65		100	16 ± 0.76		100	12 ± 0.87
	10	17 ± 0.12		10	12 ± 0.34		10	14 ± 0.13		10	8 ± 0.81
	1	7 ± 0.54		1	—		1	7 ± 0.34		1	16 ± 0.34

## Data Availability

Data is available on request.
